# The Recognition of Illegal Practice

**Published:** 1896-11

**Authors:** J. Douglas Westervelt

**Affiliations:** Alice, Texas


**The Recognition of Illegal Practice.**

*Editor Texas Medical Journal:*

I quite agree with the Journal in your late editorial on the "Medical Law," but even though wc do take the necessary steps toward the prosecution of violators of the law, they will be upheld by *some* physicians in another community into which they may move. There has been presented to my personal observation, much to my disgust, physicians in good standing, holding consultation with so-called M. D.'s whose advice cannot be given in intelligible English, and their knowledge of medicine, even more limited than is that possessed by a Schrader, the Divine Healer (?) or Don Pedrito, the Mexican "curendaro." We must say, though, that these two are more worthy, from one standpoint, than our *would be* competitors in the profession, as they remain in their places proclaimed to the world as "quacks" not looking for recognition, while the unlawful practitioners, some holding no diplomas at all, others having those from unrecognized medical schools, are consulted, and their advice accepted by doctors whose reputation extends over the State; *and who are members* of medical boards; and *this is the cause* of the tardiness manifested by many of the physicians in reporting unlawful practice. I think that a reformation should be brought about in some of *the leading physicians* of State reputation; and, too, our government authorities allow their political ribs to be tickled by such violators, appointing them to responsible State positions, thereby putting out of such positions physicians whose merits fitted them for same. This not only is injurious to the profession, but is particularly so to the poor unfortunates who have no voice in the matter. With all this before us, it is no wonder that the number of these frauds increases daily as they are encouraged by their predecessors reception into the profession.

J. DOUGLAS WESTERVELT, JR.

Alice, Texas, October 28, 1896.

